# Ethynylation of Formaldehyde over Binary Cu-Based Catalysts: Study on Synergistic Effect between Cu^+^ Species and Acid/Base Sites

**DOI:** 10.3390/nano9071038

**Published:** 2019-07-20

**Authors:** Zhipeng Wang, Lijun Ban, Pingfan Meng, Haitao Li, Yongxiang Zhao

**Affiliations:** Engineering Research Center of Ministry of Education for Fine Chemicals, School of Chemistry and Chemical Engineering, Shanxi University, Taiyuan 030006, China

**Keywords:** active cuprous species, basicity, formaldehyde ethynylation, synergistic effect

## Abstract

Most studies on the Cu-based catalysts in the ethynylation of formaldehyde are merely focused on the tuning of electronic configuration and dispersion of the Cu^+^ species. So far, little attention has been paid to the synergy between Cu species and promoters. Herein, binary nano-CuO-MO_x_ catalysts (M = Si, Al, and Mg) were synthesized and the effects of the promoter on the surface basicity/acidity were systematically studied as well as the ethynylation performance of the nano-CuO-based catalysts. The results show that the introduction of MgO provided a large number of basic sites, which could coordinate with the active Cu^+^ species and facilitate the dissociation of acetylene as HC≡C^δ−^ and H^δ+^. The strongly nucleophilic acetylenic carbon (HC≡C^δ−^) is favorable to the attack at the electropositive carbonyl C^δ+^ of formaldehyde. The MgO-promoted CuO catalyst showed the highest yield of BD (94%) and the highest stability (the BD yield decreased only from 94% to 82% after eight reaction cycles). SiO_2_ effectively dispersed Cu species, which improved catalytic activity and stability. However, the introduction of Al_2_O_3_ resulted in a large number of acidic sites on the catalyst’s surface. This led to the polymerization of acetylene, which covered the active sites and decreased the catalyst’s activity.

## 1. Introduction

1,4-butynediol (BD) contains both electron-rich –C≡C– and polar –OH groups and thus has many excellent properties. As a versatile chemical product, it is widely used in pharmaceuticals, pesticides, electroplating solutions, and the production of artificial leather [1,2,3]. More importantly, BD can be used as a critical C4 feedstock for the synthesis of the important downstream chemicals with high added value, such as 1,4-butanediol (BDO), 1,4-butenediol (BED), tetrahydrofuran (THF), γ-butyrolactone (GBL), polytetramethylene ether glycol (PTMEG), polyurethane (PU), polybutylene terephthalate (PBT), and polybutylene succinate (PBS) [4,5,6,7,8,9].

Nowadays, the Reppe method is the most widely used method for the commercial production for BD, in which formaldehyde and acetylene are subjected to a condensation reaction catalyzed by solid Cu-based catalysts [3]. Regarding the development of high-performance Cu-based ethynylation catalysts, Cu-based catalysts supported with diatomite, silica gel, and SiO_2_–MgO compound have been reported, as well as unsupported Cu-Bi nanopowder catalysts and synthetic malachite catalysts [10,11,12,13,14,15,16,17,18]. It is generally believed that these oxidized Cu-based catalysts are not active per se. In fact, it is the active cuprous species (cuprous acetylide) formed in situ during the reaction that played the catalytic role. The transition of the oxidized Cu-based precursor into the active phase involves complex physicochemical changes, including the reduction of Cu^2+^ to Cu^+^ in the formaldehyde/acetylene atmosphere and carbonization of Cu^+^ with acetylene [3,10]. Due to the complex phase transition of the Cu-based catalysts during the reaction, studies on the relationship between the structure and performance of Cu-based catalysts still remains challenging.

In recent years, our research group has carried out in-depth research into the design and catalytic mechanisms of new Cu-based ethynylation catalysts, such as CuO-Bi_2_O_3_ nano-catalyst, CuO-Bi_2_O_3_/SiO_2_-MgO aerogel catalyst, CuO-Bi_2_O_3_-Fe_3_O_4_/SiO_2_ magnetic catalyst, CuO-Bi_2_O_3_@SiO_2_ core-shell catalyst, and Cu_2_O/TiO_2_ [19,20,21,22,23,24,25]. Whether the addition of promoters or the adoption of different preparation methods or the use of different supports [19,20,21,22,23,24,25], these methods regulated the electronic configuration and dispersion of the Cu species by constructing the heterogeneous interfaces between the active components and the supporters or promoters. Nevertheless, it is well known that the supporters or promoters components at the heterogeneous interfaces can not only affect the physiochemical properties of the active sites themselves, but also coordinate with the active sites, which has always been the focus of academic research. Therefore, in order to further unfold the catalytic mechanism of ethynylation reaction, it is very necessary to study the synergistic effect between the active Cu species and other components.

Kinetic studies [26] have shown that the adsorption and activation of formaldehyde and acetylene on the surface of the catalyst critically affect the rate of ethynylation. From a theoretical perspective, the adsorption and activation of acetylene or formaldehyde through the introduction of promoters into the catalyst may alter the ethynylation performance of the catalyst. For the reactants in the ethynylation reaction of formaldehyde, the terminal ≡C–H of the acetylene molecule is acidic, while the O in the C=O unit of the formaldehyde molecule has two lone pairs of electrons. Based on these characteristics of the reactant molecules, homogeneous-phase catalysts such as NaOH, KOH, organic Li, and Lewis acids have successfully been used to prepare alkynals by reacting acetylene with carbonyl compounds bearing a variety of functional groups [27,28,29]. However, in the Cu-based heterogeneous catalytic systems, the adsorption and activation of acetylene or formaldehyde by the introduction of promoters, and synergistic catalysis involving active cuprous species during the reaction of formaldehyde with acetylene has not been reported.

Based on the above discussion, the model binary nano CuO-based catalysts doped with a variety of promoters, namely SiO_2_, Al_2_O_3_, and MgO with large differences in surface basicity/acidity, were synthesized by the ultrasound-assisted co-precipitation method. The effects of the promoter on the texture, structure, surface property, and ethynylation activity of the nano CuO-based catalysts were studied, and the structure-performance relationships of the catalysts were analyzed, which provided important inspiration for the development of highly active ethynylation catalysts.

## 2. Materials and Methods

### 2.1. Catalyst Preparation

A catalyst with a 0.84:0.16 Cu/M (M = Si, Al, or Mg) molar ratio was prepared by ultrasound-assisted co-precipitation. In a typical preparation, copper nitrate (0.084 mol) was mixed with magnesium nitrate (0.016 mol) to produce 200 mL of a mixed aqueous solution. Urea (100 g) and polyethylene glycol-400 (PEG-400, 100 mL) were sequentially added to produce a blue solution. Aqueous NaOH (200 mL, 2.4 mol/L) was added to the above solution at 80 °C with stirring to produce a black precipitate. The mixture was stirred at 80 °C for further 10 min, after which it was centrifuged and washed several times with distilled water and ethanol. The product was dried in a vacuum drying chamber at 60 °C for 24 h and calcined at 450 °C for 3 h to yield the Cu_0.84_Mg_0.16_ catalyst. Similarly, Cu_0.84_Al_0.16_ and Cu_0.84_Si_0.16_ samples were prepared with aluminum nitrate or tetraethoxysilane instead of magnesium nitrate. The Cu:M (M = Al, Si, Mg) molar ratio in Cu_0.84_M_0.16_ was close to the theoretical value of 0.84:0.16, as shown in Table 1.

For comparison, a pure CuO sample was also prepared by the above co-precipitation method without the addition of a promoter. 

### 2.2. Characterization Methods

The contents of metal elements were determined by inductively coupled plasma atomic emission spectroscopy (ICP-AES, Perkin Elmer Optima 7300 DV, Waltham, MA, USA).

Powder X-ray diffraction (XRD) patterns of the catalysts were acquired on a Bruker D8 Advance diffraction spectrometer (Karlsruhe, Germany) with Cu Kα radiation (λ = 0.154 nm). Data were recorded in the 10–80° 2*θ* range at a 2.4°/min scan rate.

N_2_-physisorption experiments were performed on a Micromeritics ASAP-2020 apparatus (Norcross, GA, USA). Specific surface area (S_BET_) was calculated using the multi-point Brunauer–Emmett–Teller (BET) procedure.

Transmission electron microscopy (TEM) was carried out using a Jeol JEM-2100 transmission electron microscope (Tokyo, Japan) operated at 200 kV.

The catalysts were inspected with X-ray photoelectron spectroscopy (XPS) on an ESCALAB 250 spectrometer (Waltham, MA, USA) using Al Kα radiation with a pass energy of 50.0 eV. The carbonaceous C1s signal at 283.1 eV was used to calibrate the binding energies.

Hydrogen temperature-programmed reduction (H_2_-TPR) experiments were performed on a Micromeritics AutoChemII 2920 instrument (Norcross, GA, USA). A mixture of 5 vol% H_2_ in N_2_ (30 mL min^−1^) was introduced, and the temperature was raised from room temperature to 500 °C at 10 °C min^−1^.

NH_3_ and CO_2_ temperature-programmed desorption (TPD) profiles were acquired on the same apparatus. Samples (100 mg, 40–60 mesh) were pretreated at 500 °C under a flow of N_2_ at a rate of 60 mL min^−1^ for 60 min and saturated with a flow of pure NH_3_ or CO_2_ after cooling to 100 °C. To remove physiosorbed NH_3_ or CO_2_, these pre-treated samples were then purged in a helium atmosphere at 100 °C until the baseline was stable, after which they were heated from 100 °C to 600 °C at 10 °C min^−1^ under a flow of helium. The amount of NH_3_ or CO_2_ evolved from the sample was determined using a thermal conductivity detector (TCD).

UV-Raman spectra were recorded with a LabRAM HR Evolution instrument (Kyoto, Japan) equipped with a charge coupled device (CCD) detector at room temperature. A 325 nm Ventus laser operating at 10 MW was used as the excitation source.

Pyridine-infrared (Py-IR) spectroscopy was performed using an in situ vacuum adsorption infrared characterization system at the Dalian Institute of Chemical Physics (Dalian, China). Self-supporting tablet samples (20 mg) were placed in an in situ reaction cell, pretreated in a vacuum at 120 °C for 90 min, allowed to adsorb pyridine at room temperature, treated in a vacuum again at 100 °C for 30 min, and then allowed to return to room temperature. After this, an IR spectrum was recorded using a Bruker Tensor 27 Fourier-transform infrared spectrometer. 

CO/C_2_H_2_-adsorbed infrared (CO/C_2_H_2_-IR) spectra of the catalysts were recorded using the same apparatus equipped with a highly sensitive MCT detector cooled by liquid N_2_. The used samples were pre-treated at 60 °C for 3 h. CO/C_2_H_2_ gas was introduced into the system for 30 min, after which it was expelled.

### 2.3. Evaluating Catalytic Activity

2.5 g of the catalyst and 50 mL of aqueous formaldehyde (35 vol%) were sequentially added to a 100-milliliter round-bottom three-port flask equipped with a thermometer and a condenser tube. The flask was purged with N_2,_ and the system was heated to 90 °C in an oil bath with stirring, after which C_2_H_2_ gas was introduced for 24 h. In this evaluation, the C_2_H_2_ flow was excessive. The mixture was cooled to room temperature after the reaction. The solid catalyst was removed by centrifugation and quantitatively analyzed on an Agilent 7890A gas chromatograph fitted with a DB-5 capillary column (0.32 mm × 50 m) and an flame ionization detector (FID). 1,4-butanediol was used as the internal standard. The unconverted formaldehyde in the solution was determined by iodimetry [19,20].

## 3. Results

### 3.1. Structure, Texture, and Surface Valence State of the Catalysts

**Figure 1:**
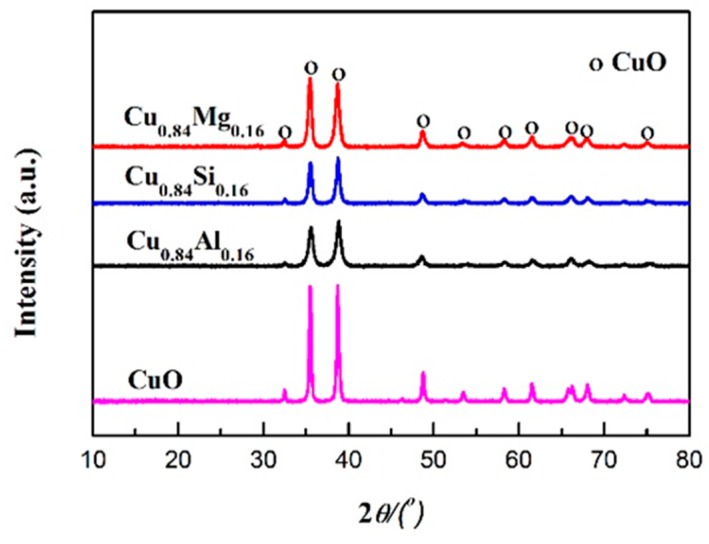
X-ray diffraction (XRD) patterns of the catalysts.

Figure 1 shows the XRD patterns of pure CuO and Cu_0.84_M_0.16_ catalysts doped with different promoters. Each catalyst exhibited characteristic diffraction peaks of the (110), (002), (111), (−202), (020), (202), (−113), (−311), (220), and (004) crystal planes of CuO (JCPDS card No. 48-1548) at *2θ* values of 32.6°, 35.5°, 38.7°, 48.8°, 53.4°, 58.3°, 61.7°, 66.2°, 68.1°, and 74.9°, respectively. Cu_0.84_M_0.16_ exhibited no obvious promoter diffraction peaks, indicating the promoters were present in amorphous states or highly dispersed at levels below the XRD detection limit (2–5 nm). Using the Scherrer formula (D = Kλ/Bcos*θ*) and the data that include the *2θ* values and half-peak widths of the CuO (111) crystal planes, the CuO-grain sizes in Cu_0.84_Al_0.16_, Cu_0.84_Si_0.16_, Cu_0.84_Mg_0.16_, and pure CuO were calculated to be 15.8 nm, 14.9 nm, 18.6 nm, and 22.3 nm, respectively (Table 1). This suggests that the introduction of the promoter contributed to the CuO dispersion and inhibited the aggregation and growth of CuO grains during calcination. Among the three introduced promoters, CuO dispersed by Al and Si was more pronounced due to the high thermal stability of Al_2_O_3_ and SiO_2_. The texture parameters of Cu_0.84_M_0.16_ and pure CuO are listed in Table 1. Compared with pure CuO, the specific surface area and pore volume of Cu_0.84_M_0.16_ increased to varying degrees, indicating that the introduction of promoters not only dispersed CuO but also facilitated the development of its pore structure.

**Figure 2:**
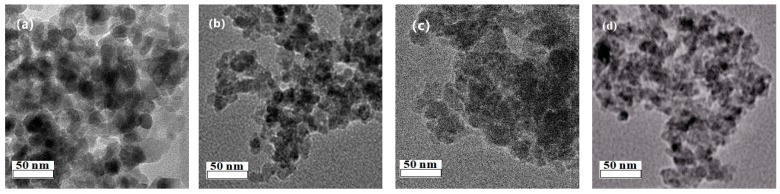
TEM images of (**a**) CuO, (**b**) Cu_0.84_ Si_0.16_, (**c**) Cu_0.84_Mg_0.16_, and (**d**) Cu_0.84_Al_0.16_.

Figure 2 shows the TEM image of each catalyst. The CuO particles in the pure CuO sample are irregular, round, and about 20–25 nm in size. After the introduction of promoters, by contrast, the particle size of CuO decreased in varying degrees. These observations indicate that the introduced promoters, particularly Al and Si, played a good dispersive role and inhibited the agglomeration of particles during the catalyst preparation, which are consistent with those of XRD.

**Figure 3:**
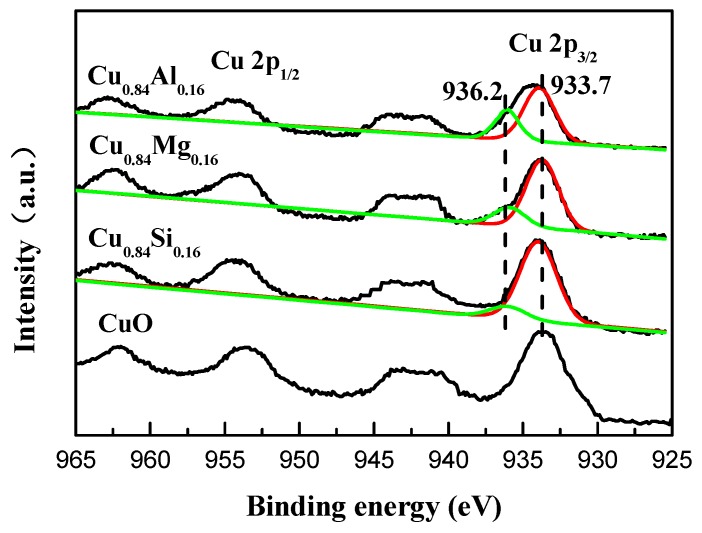
Cu 2p X-ray photoelectron spectroscopy (XPS) spectra of the catalysts.

Figure 3 shows the Cu 2p XPS spectra of pure CuO and Cu_0.84_M_0.16_ samples. The peak located at 933.5 eV was ascribed to characteristic Cu 2p_3/2_ and the one at 953.7 eV to Cu 2p_1/2_ for Cu^2+^ of CuO [30,31]. Considering the asymmetry of the Cu2p_3/2_ envelope, these peaks could be deconvoluted into two peaks centered at around 933.7 eV and 936.2 eV, which implies the existence of two Cu^2+^ species in different chemical environments. The former corresponds to free CuO species, while the latter belongs to CuO with strong interaction with promoters [30,31,32]. The higher binding energy (936.2 eV) suggests a charge transfer from Cu^2+^ ions toward the promoter [32]. Notably, the 936.2 eV peak in the spectrum of Cu_0.84_Al_0.16_ is apparently the strongest among the catalysts, which is possibly due to stronger interactions between CuO and Al_2_O_3_ than those involving other promoters.

### 3.2. Reduction Properties of the Catalysts

**Figure 4:**
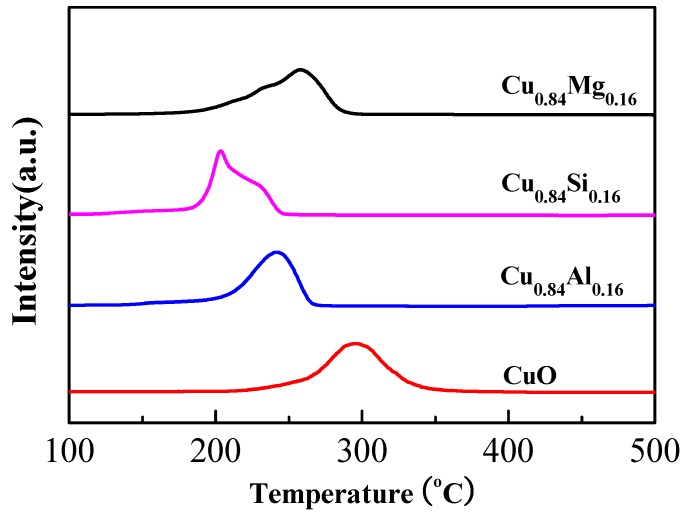
H_2_- temperature-programmed reduction (TPR) profiles of the catalysts.

H_2_-TPR tests of each catalyst were performed and displayed in Figure 4. Since MgO, Al_2_O_3_, and SiO_2_ are extremely difficult to reduce in an H_2_ atmosphere, the hydrogen consumption peaks of each sample correspond to the reduction of CuO to metallic Cu. Pure CuO exhibited a symmetrical reduction peak at ~295 °C, whereas the reduction peaks of doped Cu_0.84_M_0.16_ were observed at lower temperatures. Cu_0.84_Mg_0.16_ exhibited the main hydrogen-consumption peak at 260 °C. The reduction peak of Cu_0.84_Al_0.16_ appeared at 240 °C. Cu_0.84_Si_0.16_ showed the lowest reduction-peak temperature, at about 200 °C. Obviously, the reduction-peak temperatures for all samples followed the order: CuO > Cu_0.84_Mg_0.16_ > Cu_0.84_Al_0.16_ > Cu_0.84_Si_0.16_, which was approximately consistent with the order of CuO grain sizes shown by the XRD data, namely: CuO > Cu_0.84_Mg_0.16_ > Cu_0.84_Al_0.16_ ≈ Cu_0.84_Si_0.16_. This is roughly consistent with the literature reports that the larger the grain size of CuO, the higher the reduction temperature [33,34]. It’s worth noting that the CuO grain size of Cu_0.84_Al_0.16_ and Cu_0.84_Si_0.16_ was similar, but Cu_0.84_Al_0.16_ exhibited a higher reduction temperature than Cu_0.84_Si_0.16_. According to the XPS results, this is mainly due to stronger interactions between CuO and Al_2_O_3_, which inhibit the reduction of CuO [35].

### 3.3. Acidic and Basic Properties of the Catalysts

**Figure 5:**
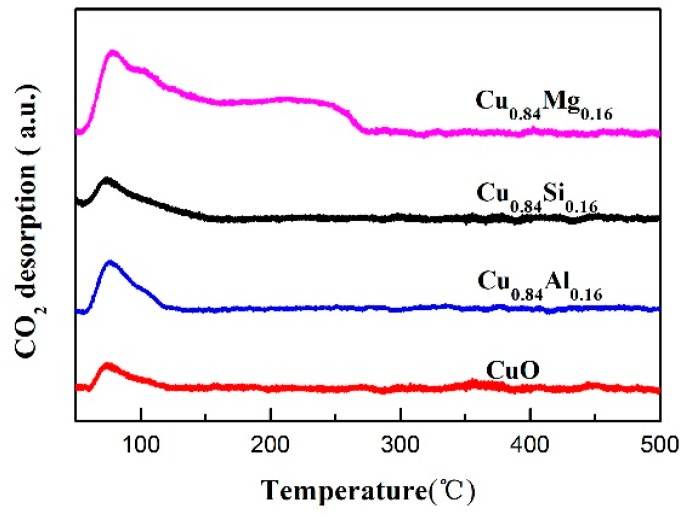
CO_2_- temperature-programmed desorption (TPD) profiles of the catalysts.

Figure 5 shows the CO_2_-TPD results for each catalyst. It can be seen that pure CuO, Cu_0.84_Si_0.16_, and Cu_0.84_Al_0.16_ showed only a weak CO_2_-desorption peak at around 90 °C, indicating that there were almost weak basic sites on their surface [36]. In contrast, in addition to the CO_2_-desorption peak at around 90 °C, a broad CO_2_ desorption platform is visible in the 150–260 °C region of the CO_2_-TPD profile of Cu_0.84_Mg_0.16_, which corresponds to medium-strong basic sites [36] and indicates that Mg doping significantly increased the strength and number of basic sites on the catalyst’s surface. The XRD results for this catalyst showed that MgO was poorly crystalline and amorphous. The surface also had more defect sites, such as edges, angles, and steps, which led to more unsaturated O^2−^ sites on the surface and enhanced its basicity [37]. The values of the basicity calculated from the CO_2_-TPD profiles were listed in Table 2.

**Figure 6:**
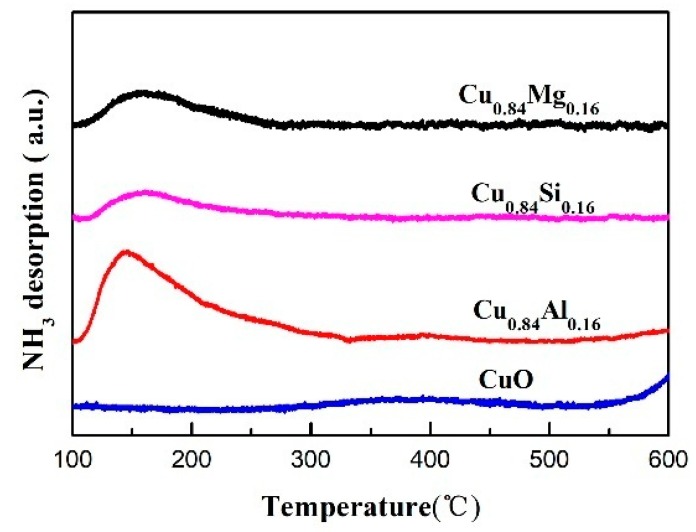
NH_3_-TPD profiles of the catalysts.

**Figure 7:**
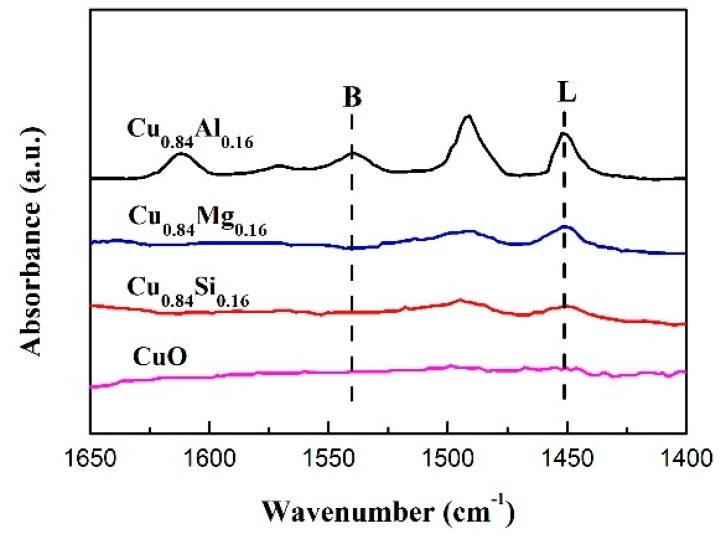
In situ Py-IR spectra of the catalysts.

Figure 6 displays the NH_3_-TPD results for each catalyst. No obvious desorption peak is observed in the 100–600 °C temperature range in the profile of pure CuO, which indicates that there is almost no acidity on the surface. Cu_0.84_Si_0.16_ and Cu_0.84_Mg_0.16_ exhibited weak NH_3_ desorption peaks between 100 °C and 200 °C, consistent with weakly acid sites on the surface of these catalysts [38]. In contrast, Cu_0.84_Al_0.16_ exhibited a broad NH_3_ desorption peak between 100 °C and 400 °C in its NH_3_-TPD profile; the intensity and area of this peak are significantly greater than those of other samples, indicating that medium and strong acidic sites exist on the surface of this sample [39]. It can be seen from Table 2 that the total acid amount of Cu_0.84_Al_0.16_ was up to 69.6 μmol·g^−2^. The Py-IR spectra of the catalysts (Figure 7) exhibited spectral bands at around 1450 cm^−1^, which corresponds to pyridines at the Lewis acid sites, and 1540 cm^−1^, which corresponds to pyridinium ions at the Brønsted acid sites. The band at 1490 cm^−1^ is attributed to the contribution of pyridines interacting with both Lewis- and Brønsted-acidic sites [40]. 

As the CuO sample exhibited almost no surface acidity, the presence of both L and B acid sites on Cu_0.84_Al_0.16_ surface indicate that the surface acidity of the Cu_0.84_Al_0.16_ sample is mainly due to Al_2_O_3_. The Lewis-acidic and Brønsted-acidic sites may have originated from the surfaces of the unsaturatedly coordinated Al^3+^ and Al–OH interactions with the Cu species [41], respectively. In contrast, Cu_0.84_Si_0.16_ and Cu_0.84_Mg_0.16_ have only small amounts of Lewis acid sites on their surfaces, and no obvious acidic sites were detected for the CuO sample. These results are consistent with those obtained from the NH_3_-TPD experiments.

### 3.4. Structure, Texture, and Surface Analysis of the Catalysts After the Reaction

Cuprous species (cuprous acetylide) are considered to be the active species in formaldehyde ethynylation. In general, cuprous acetylide is formed in situ during the reaction, which includes the reduction of Cu^2+^ to Cu^+^, followed by the complexation of Cu^+^ with acetylene to form cuprous acetylide [19,20]. The changes in the structure, texture, and surface properties of activated catalysts were characterized by XRD, N_2_-physisorption, Raman, and CO-IR methods.

**Figure 8:**
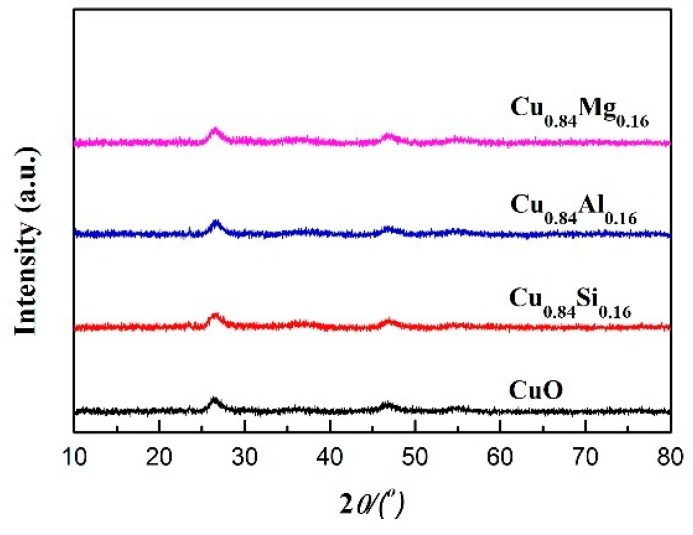
XRD patterns of used catalysts.

Figure 8 shows the XRD pattern of each catalyst after 15 h of evaluation (i.e., following activation). Compared with fresh catalysts, the CuO characteristic diffraction peaks disappeared, whereas diffused diffraction peaks at *2θ* values of 26.3° and 46.8° were observed in used catalysts. This result indicates that CuO was transformed into a new species through a phase change, and the new species is presumed to be an amorphous active cuprous acetylide. No excessive reduction product—metallic Cu—was observed in any of the samples.

The phase transformation of CuO further resulted in changes of the catalyst texture. As summarized in Table 1, the specific surface area and pore volume of the used CuO catalyst decreased significantly (~70%) compared to those of the fresh catalyst. This can be ascribed to the collapse of porous channels and overall skeletal shrinkage during phase transformation. The catalysts doped with promoter exhibited less significant decreases in specific surface area (approximately 15–45%). This was due to the introduction of SiO_2_, Al_2_O_3_, and MgO, which effectively disperse Cu species and support the formed cuprous species. 

**Figure 9:**
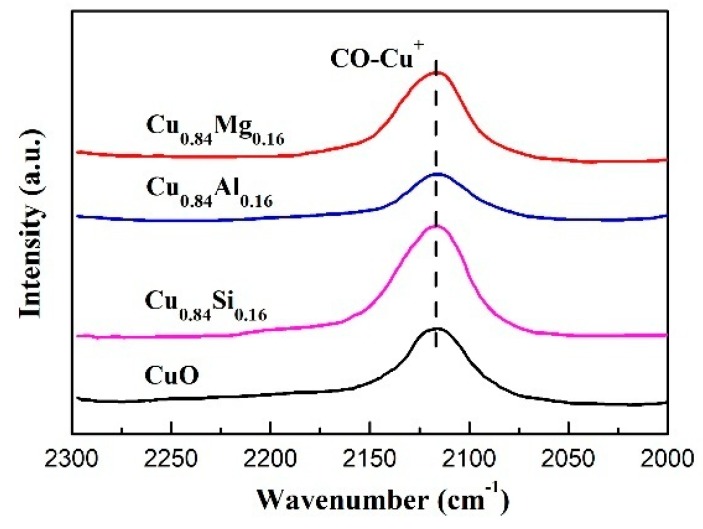
CO-IR spectra of used catalysts.

Cu^+^ in cuprous acetylide is generally considered as the active center of formaldehyde ethynylation. Hence, the amount of exposed Cu^+^ on the catalyst surface is an important factor [19,20]. By using XPS and Cu LMM Auger (Figure S1), we have proved that only Cu^+^ species existed on the used catalysts. Owing to the strong chemisorption of CO by Cu^+^ at room temperature, CO-IR spectroscopy was used to characterize the Cu^+^ species on the surface of used catalysts. As it is evident from Figure 9, only one infrared peak at 2137 cm^−1^ was observed in the spectrum of each catalyst, which was attributable to the linear adsorption of CO molecules on the Cu^+^ sites [42,43,44,45,46]. Based on the semi-quantitative method reported in the literature [47], the integral area of the CO-Cu^+^ IR peaks of the catalysts (denoting the amount of Cu^+^ exposed on the catalyst surface) increased in the order: Cu_0.84_Al_0.16_ < CuO < Cu_0.84_Mg_0.16_ < Cu_0.84_Si_0.16_. 

The amount of Cu^+^ exposure in a used catalyst is affected by many factors, such as the grain size of the initial CuO species, physical specific surface area, and coverage of inactive species. According to the XRD patterns of fresh catalysts and the N_2_-physisorption results of used catalysts, the CuO grain size followed the order: Cu_0.84_Si_0.16_ ≈ Cu_0.84_Al_0.16_ < Cu_0.84_Mg_0.16_ < CuO. The specific surface area of used catalysts was: Cu_0.84_Si_0.16_ > Cu_0.84_Al_0.16_ > Cu_0.84_Mg_0.16_ > CuO. In general, small CuO grains and a large specific surface area are favorable to obtaining highly dispersed Cu^+^ species. All studied catalysts except Cu_0.84_Al_0.16_ adhered to this rule. Pure CuO showed the largest grain size and the smallest specific surface area after the reaction, resulting in less Cu^+^ exposure, whereas Cu_0.84_Si_0.16_ had small grain size and the largest specific surface after the reaction, resulting in the highest Cu^+^ exposure. However, Cu_0.84_Al_0.16_ exhibited the lowest number of exposed Cu^+^ sites despite having similarly sized CuO grains to that of Cu_0.84_Si_0.16_ and a high specific surface area; we speculate that the generated Cu^+^ sites might be covered by inactive species in this sample. 

**Figure 10:**
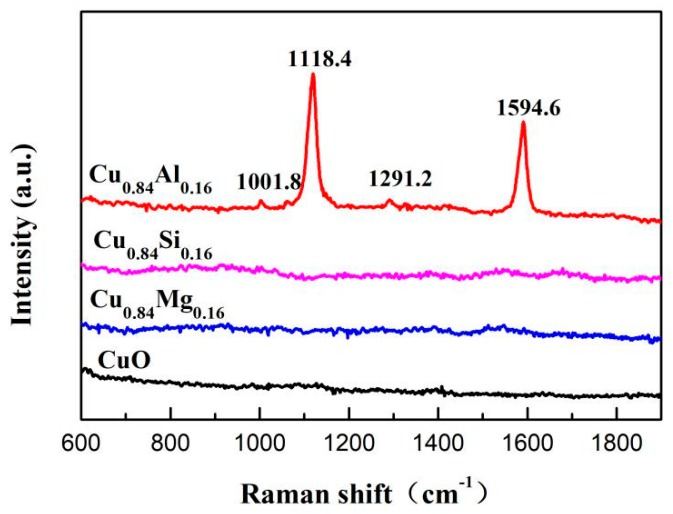
Raman spectra of used catalysts.

To obtain the surface species on the catalysts, UV-Raman spectroscopy was carried out. As shown in Figure 10, Cu_0.84_Al_0.16_ exhibited four Raman peaks at 1001.8 cm^−1^, 1291.2 cm^−1^, 1118.4 cm^−1^, and 1594.6 cm^−1^, which are attributed to polyacetylene [48,49], indicating that this polymer is formed on the surface of the catalyst during the reaction. Polyacetylene coverage decreased the Cu^+^ exposure in used Cu_0.84_Al_0.16_. In general, two factors control the formation of polyacetylene: (1) over-reduction of Cu^2+^ to Cu provides a catalyst that facilitates acetylene polymerization [3,19], and (2) acidic centers on the catalyst surface favor the formation of polyacetylene [50,51]. Peaks corresponding to metallic Cu were not observed in the XRD patterns of used catalysts. However, medium-strong acidic sites on the surface of Cu_0.84_Al_0.16_ were observed by NH_3_-TPD and Py-IR spectroscopy. These acidic sites may be responsible for promoting the formation of polyacetylene that covers the surface of the catalyst, resulting in decreased Cu^+^ exposure. 

### 3.5. Catalytic Performance

The main reaction formula of formaldehyde ethynylation can be expressed as follows:

HC≡CH + 2HCHO → HO–CH_2_C≡CCH_2_–OH

**Figure 11:**
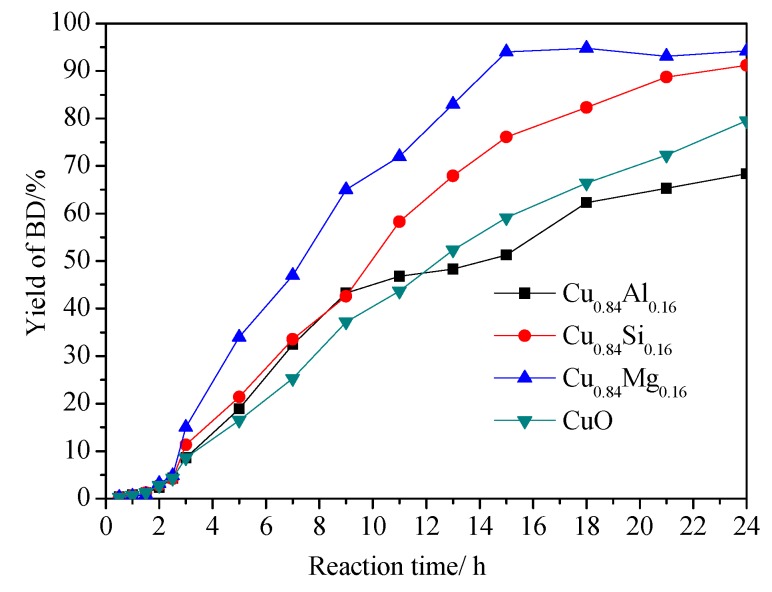
The yield of 1,4-butynediol (BD) by CuO and Cu_0.84_M_0.16_ catalysts. Reaction conditions: Catalyst amount, 2.5 g; formaldehyde solution concentration, 35 vol.%; consumption of the formaldehyde solution, 50 mL; reaction temperature, 90°C.

This reaction plays a pivotal role in connecting coal-based primary chemicals (acetylene and formaldehyde) and high value-added chemicals (BD and its downstream chemicals), which improves the efficient and comprehensive utilization of coal resources. Thus, it is of great industrial importance to design and develop ethynylation catalysts. In the present work, the ethynylation reaction of formaldehyde to produce BD was performed over these prepared Cu_0.84_M_0.16_ catalysts in a simulated slurry reactor at 90 °C and under atmospheric pressure. Only BD was observed in the gas chromatography analysis of reaction products, and no other by-products were found. Therefore, the yield of BD was used to reflect the activity of the catalyst. As shown in Figure 11, the yield of BD varied with the increasing reaction time. It can be seen that an obvious induction period took place in the first 2 h of the reaction because of the phase transformation of CuO precursors. After the induction period, the yield of BD increased with the reaction time for all catalysts. Only in 15 h, the yield of BD by Cu_0.84_Mg_0.16_ reached a maximum of 94%. Cu_0.84_Si_0.16_ showed lower activity than Cu_0.84_Mg_0.16_, with a BD yield of approximately 91% at 24 h. On the other hand, the BD yields at 24 h were only about 79% for CuO and 68% for Cu_0.84_Al_0.16_, which is significantly lower than the yields of Cu_0.84_Mg_0.16_ and Cu_0.84_Si_0.16_. In addition, none of the promoters showed the ethynylation activity, which therefore were not presented in Figure 11.

**Figure 12:**
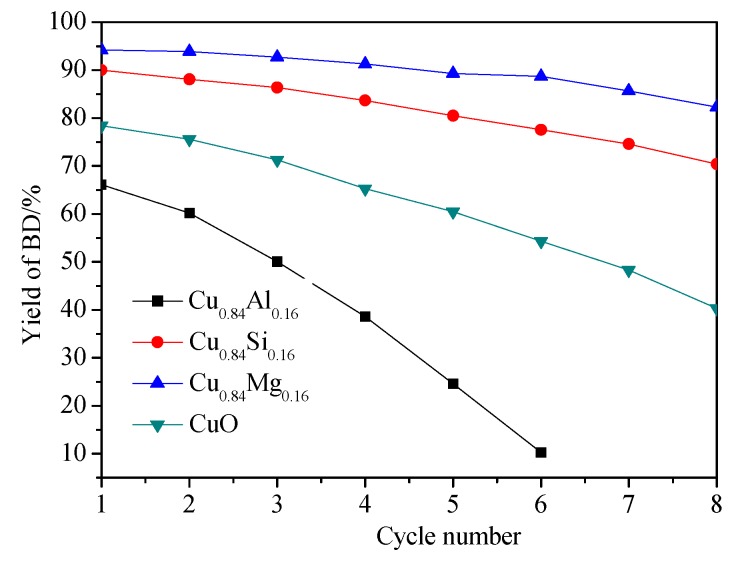
The yield of BD by CuO and Cu_0.84_M_0.16_ catalysts as a function of the cycle number. Reaction conditions: Catalyst amount, 2.5 g; formaldehyde solution concentration, 35 vol.%; consumption of the formaldehyde solution, 50 mL; reaction temperature, 90°C, reaction time, 24 h.

Since stability is critical for practical application of catalysts for formaldehyde ethynylation, the eight cycles of evaluation over the prepared catalysts were investigated. The results of the stability evaluation of CuO and Cu_0.84_M_0.16_ catalysts are shown in Figure 12. During six reaction cycles, a drastic decrease in BD yield from 66% to 10% was observed for Cu_0.84_Al_0.16_, which was apparently lower than that of other catalysts. In the next two cycles, the Cu_0.84_Al_0.16_ catalyst lost its activity completely. However, compared to Cu_0.84_Al_0.16_, Cu_0.84_Si_0.16_ and Cu_0.84_Mg_0.16_ showed better ethynylation activity; the BD yield of Cu_0.84_Si_0.16_ and Cu_0.84_Mg_0.16_ decreased from 90% to 70% and 94% to 82%, respectively. 

The above results of the catalytic performance suggest that CuO doped with different promoters influenced the activity and stability to varied degree, while the texture and surface property of the catalysts was closely related to the evaluation, which will be discussed in detail in the next section.

## 4. Discussion

**Figure 13:**
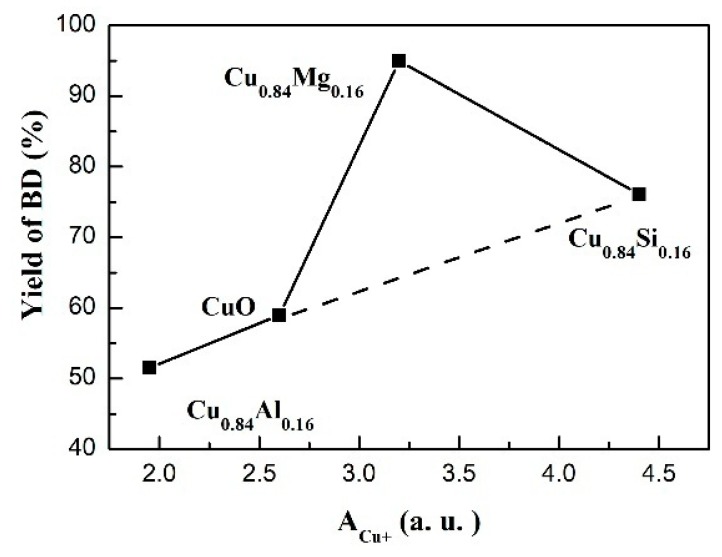
The BD yield of catalysts after 15 h as a function of the integrated area of the Cu^+^ peak (A_Cu+_) determined by CO-IR spectroscopy.

In this study, SiO_2_, Al_2_O_3_, and MgO were chosen as the promoter for copper-based catalysts. Different promoters influence not only the physicochemical nature but also the ethynylation performance. For ethynylation of formaldehyde, it is accepted that Cu^+^ species act as the main active sites [19,20]. Our previous work [19] pointed out that Cu^+^ is related to the adsorption and activation of reactant molecules of acetylene and formaldehyde. The number of exposed Cu^+^ sites on the surface of the catalysts are responsible for the ethynylation performance. CO-IR results of the used catalysts provided us the numeric expressions about the exposed Cu^+^ sites. Associating the amount of the exposed Cu^+^ with the corresponding BD yield in 15h, an evident linear relationship was observed except for Cu_0.84_Mg_0.16_ (Figure 13), indicating that Cu^+^ species are the only active sites of the ethynylation process for CuO, Cu_0.84_Si_0.16_ or Cu_0.84_Al_0.16_.

Compared with pure CuO, Cu_0.84_Si_0.16_ showed smaller CuO grain size, higher CuO dispersion, and suitable reduction property (Figure 1, Figure 2, and Figure 4), because of the dispersive and supportive function of SiO_2_. In the ethynylation of formaldehyde, the highly dispersed CuO was effectively transformed into the highly dispersed cuprous acetylide species without the production of inactive metallic Cu, exposing more Cu^+^ centers (Figure 9) on the surface of the catalyst, and thus enabling Cu_0.84_Si_0.16_ to exhibit higher ethynylation activity than that of CuO (Figure 11). Meanwhile, the dispersive and supportive function of SiO_2_ effectively prevent the migration and aggregation of the active species during the cyclic experiment, which improved the catalyst’s stability.

As with the similar XRD, BET, and TEM results of CuO doped with SiO_2_, Al_2_O_3_ also played a role in dispersing CuO in Cu_0.84_Al_0.16_, which is favorable to obtaining highly dispersed active species and results in a large amount of exposed Cu^+^. It is clear from the evaluation data in Figure 11 that the increased BD yield of Cu_0.84_Al_0.16_ was close to that of Cu_0.84_Si_0.16_ and higher than that of CuO in the first 10 h of the reaction. However, with the prolongation of reaction time, the BD yield of Cu_0.84_Al_0.16_ just increased slightly, finally reaching lower values than that of CuO and Cu_0.84_Si_0.16_. We tried to explain the possible reason to account for this phenomenon. The migration and aggregation of the active Cu^+^ species can be first ruled out due to the dispersive and supportive function of Al_2_O_3_. On the other hand, according to the study of Bordiga et al. [50] and Cox et al. [51], acetylene and methylacetylene can be interacted with Brønsted acid centers on hydrogen zeolites to produce a series of linear polyenes. In this work, due to the NH_3_-TPD and Py-IR results of Cu_0.84_Al_0.16_, it can be seen that there is a large number of Lewis (Al^3+^ with unsaturated coordination) and Brønsted (Al-OH) acid sites on Cu_0.84_Al_0.16._ In aqueous formaldehyde solution at 90 °C (the actual reaction condition), some of the Al^3+^ Lewis acid sites might convert to Al-OH by hydration with H_2_O molecules. Thus, it can be inferred that, during the reaction, these Brønsted acid center on Cu_0.84_Al_0.16_ could promote the polymerization of acetylene into polyacetylene. The UV-Raman spectra of Cu_0.84_Al_0.16_ used for 24 h in the ethynylation of formaldehyde showed the presence of polyacetylene on the surface, further confirming this inference. Polyacetylene covered the active Cu^+^ center, which resulted in decreased catalytic activity and rapid deactivation of the catalyst during recycling.

**Figure 14:**
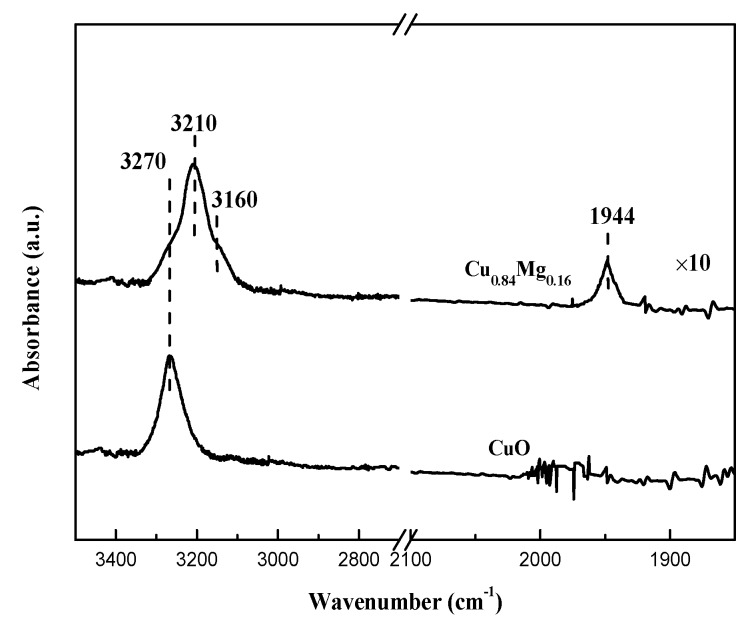
C_2_H_2_-IR spectra of catalysts after reaction.

Cu_0.84_Mg_0.16_ showed the highest catalytic activity which apparently deviates the linear relationship between the amount of exposed Cu^+^ and the BD yield (Figure 13), suggesting other factors besides the active Cu^+^ species could affect the catalytic activity of the Cu_0.84_Mg_0.16_. Based on the systematic characterization results of the Cu_0.84_Mg_0.16_, the introduction of MgO improved the dispersion of the copper species, but more importantly, it enhanced the basicity of the catalysts. It is widely accepted that basic sites could activate sp C–H to produce HC≡C^δ−^ and H^δ+^ [52]. Therefore, in theory, the medium-strong basic sites on the Cu_0.84_Mg_0.16_ surface might play a role in assisting the active Cu^+^ species to activate the C_2_H_2_ molecules. In order to clarify adsorption state of C_2_H_2_ on catalysts, in-situ C_2_H_2_-FTIR was exploited to study the chemical state of C_2_H_2_ on the used CuO and Cu_0.84_Mg_0.16_. The normal modes of gaseous C_2_H_2_ in IR spectrum are listed as follows: IR-inactive ν_1_ (sym. ≡C–H) at 3372 cm^−1^, IR-inactive ν_2_ (C≡C) at 1974 cm^−1^, IR-active ν_3_ (asym. ≡ C–H) at 3287 cm^−1^ [53]. However, the forbidden ν_2_ mode could be activated by the adsorption on catalysts surface [53,54]. As shown in Figure 14, the adsorption of acetylene on CuO leads to the appearance of an intense asymmetric ≡ C–H band at 3270 cm^−1^ (Δν = 17 cm^−1^), which is caused by the interaction between coordinatively unsaturated Cu^+^ cations and the triple bond of acetylene [55,56]. No obvious band for the C≡C bond indicates the symmetrical coordination of acetylene. For Cu_0.84_Mg_0.16_, the asymmetric ≡ C–H band shifted to 3210 cm^−1^ (Δν = 77 cm^−1^), indicating the activation ability of C_2_H_2_ was further enhanced. This is due to the synergistic interaction of the Cu^+^ species and the nearby medium-strong basic sites [54,55,56,57]. Moreover, the high polarizability of O^2−^ sites on MgO results in the presence of the should band at 3160 cm^−1^ (asym. ≡ C–H) and the band at 1944 cm^−1^ (C ≡ C) [55]. 

**Figure 15:**
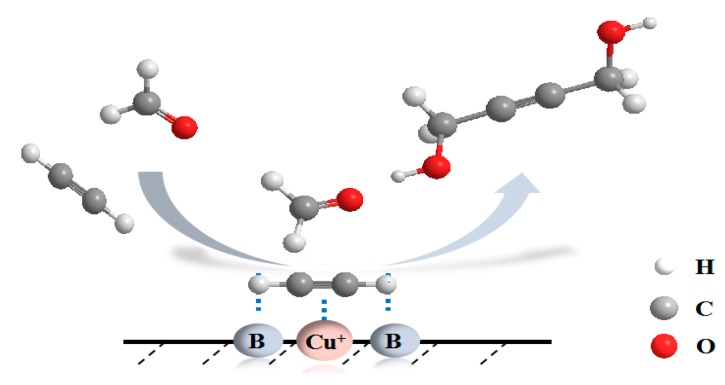
A plausible reaction mechanism on Cu_0.84_Mg_0.16_ catalyst.

On the basis of the above discussion, a plausible synergistic catalytic process for the ethynylation of formaldehyde reaction over Cu_0.84_Mg_0.16_ catalyst is tentatively proposed to rationalize the structure-performance relationship. As shown in Figure 15, the active Cu^+^ species and the basic site act on C≡C and H of acetylene, respectively, which enhances the nucleophilicity of acetylenic carbon. A strongly nucleophilic acetylenic carbon (HC≡C^δ−^) is favorable to the attack at the electropositive C^δ+^ in the C=O unit of formaldehyde, which promotes the formation of BD. Basic centers help to activate acetylene molecules; hence, Cu_0.84_Mg_0.16_ exhibits optimal ethynylation performance through a synergism between basic centers and Cu^+^.

## 5. Conclusions

The dispersion of the active components in a Cu-based catalyst and their adjacent acid or base sites are key factors that affect catalytic activity and stability in the ethynylation reaction. The introduction of SiO_2_ results in the most dispersed active components, which, in turn, increase the catalytic performance. The introduction of MgO not only improves the dispersion of Cu^+^ species to some extent, but it also results in an abundance of basic sites. The synergism between the active Cu^+^ sites and basic sites greatly improves the catalytic activity and stability. The introduction of Al_2_O_3_ increases the acidity of the catalyst’s surface and results in the formation of polyacetylene during the reaction, which covered the catalyst’s surface and gave rise to the decreased catalytic activity and the rapid deactivation. This work not only clarifies the precise roles of SiO_2_, MgO, and Al_2_O_3_ during ethynylation process, but more importantly, reveals the synergistic mechanism of the Cu^+^-base sites, which introduces new guidelines for rational development of new and highly efficient Cu-based ethynylation catalysts. 

## Figures and Tables

**Table 1:** Structural and textural properties of the catalysts.

Sample	Cu/M ^a^	D_CuO_ (nm)	A_*BET*_ (m^2^/g)		V_*Total*_ (cm^3^/g)		A_Cu_+ ^b^ (a.u.)
			Fresh	Used	Fresh	Used	
CuO	--	22.3	19.7	5.2	0.10	0.03	2.6
Cu_0.84_Al_0.16_	0.82/0.18	15.8	51.1	43.6	0.27	0.23	1.95
Cu_0.84_Si_0.16_	0.85/0.15	14.9	60.4	50.8	0.29	0.25	4.4
Cu_0.84_Mg_0.16_	0.87/0.13	18.6	38.9	21.4	0.17	0.08	3.2

^a^ Cu/M molar ratios determined by ICE-AES. ^b^ A_Cu_+ values were calculated from the Cu^+^–CO peak area for 0.5 g of the catalyst.

**Table 2:** Acid-base properties of the catalysts.

Sample	CuO	Cu_0.84_Al_0.16_	Cu_0.84_Si_0.16_	Cu_0.84_Mg_0.16_
Basicity ^a^ (μmol·g^−2^)	12.8	24.7	15.3	146.4
Acidity ^b^ (μmol·g^−2^)	0.9	69.6	9.2	11.4

^a^ Basicity was calculated by CO_2_-TPD; ^b^ Acidity was calculated by NH_3_-TPD.

## Supplementary Materials

The following are available online at https://www.mdpi.com/2079-4991/9/7/1038/s1, Figure S1: XPS spectra (a) and X-ray excited Auger electron spectroscopy (XAES) spectra (b) of the catalysts after reaction after reaction.
