# Supplementary material for: Ethynylation of Formaldehyde over Binary Cu-Based Catalysts: Study on Synergistic Effect between Cu+ Species and Acid/Base Sites
Source: Nanomaterials (Basel). 2019 Jul 20;9(7):1038. doi: 10.3390/nano9071038 (PMC6669766; doi:10.3390/nano9071038)
Supplement: Supplementary file 1 [file nanomaterials-09-01038-s001.pdf]

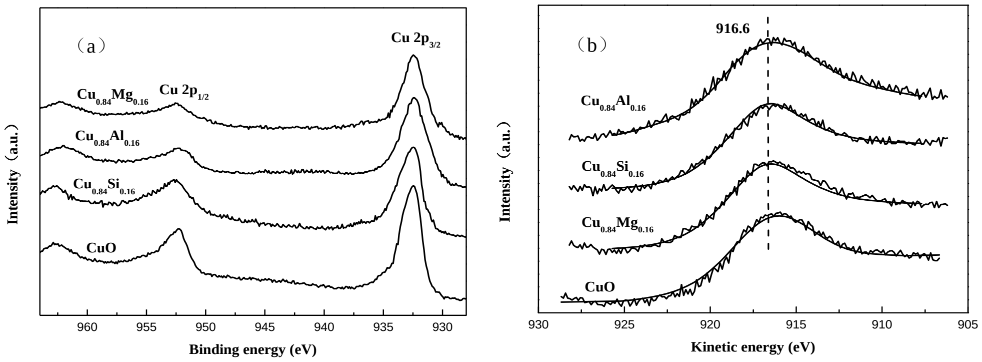

Figure S1. XPS spectra (**a**) and X-ray excited Auger electron spectroscopy (XAES) spectra (**b**) of the catalysts after reaction after reaction.

It can be seen from Figure S1a that, compared with the fresh catalysts, the BE value of $Cu2p_{3/2}$ shifts to around 932.2 eV and the satellite peak disappears. This phenomenon is due to the reduction of Cu^{2+} to Cu^{+} and/or Cu^{0} [1]. Because of the similar BE value between Cu^{+} and Cu^{0} species, it is hard to distinguish the copper species with low valence via using XPS. Therefore, the Cu LMM XAES spectrum is employed to discriminate the copper species. As displayed in Figure S1b, the symmetric Auger kinetic energy peak at around 916.6 eV corresponding to Cu^{+} species could be observed [1,2] , indicating only Cu^{+} species exits on the catalysts.

References

1. Zhu, Y.F.; Zhu, Y.L.; Ding, G.Q.; Zhu, S.H.; Zheng, H.Y.; Li, Y.W. Highly selective synthesis of ethylene glycol and ethanol via hydrogenation of dimethyl oxalate on Cu catalysts: Influence of support. *Appl. Catal. A: Gen.* **2013**, *468*, 296–304.
2. Li, H.T.; Ban, L.J.; Wang, Z.P.; Meng, P.F.; Zhang, Y; Wu, R.F.; Zhao, Y.X. Regulation of Cu species in CuO/SiO_2 and its structural evolution in ethynylation reaction. *Nanomaterials*. **2019**, *9*, 842–857.
